# Transmitting information of an object behind the obstacle to infinity

**DOI:** 10.1038/srep13140

**Published:** 2015-08-14

**Authors:** Bai Bing Xu, Wei Xiang Jiang, Ling Ling Meng, Tie Jun Cui

**Affiliations:** 1 State Key Laboratory of Millimeter Waves, Department of Radio Engineering, Southeast University, Nanjing 210096, China

## Abstract

We propose an illusion device that transforms a metallic cylinder into a Luneburg lens by using transformation optics. Such a transformed focusing lens guides electromagnetic waves to propagate around the central metallic cylinder smoothly and be focused on one spot, and thus the information of an object behind the obstacle can be transmitted to infinity. In order to realize the required-anisotropic parameters with high permittivity and low permeability, we design embedded split-ring resonators (SRRs) to increase the permittivity of the traditional SRR structures. In experiments, we fabricate and measure the transformed lens, and the tested results agree well with the numerical simulations and theoretical predictions. The proposed transformation lens can mimic some properties of Einstein gravitational lens because their wave propagation behaviors are very similar.

In the past ten years, metamaterials have attracted a lot of attention due to their novel electromagnetic properties, which can be obtained by designing special artificial structures and arranging them periodically or non-periodically. Transformation optics (TO) has provided a strategy to control electromagnetic field freely by the coordinate transformation and become a powerful tool to design anisotropic or nearly-isotropic devices^1^,^2^. Many interesting devices based on TO theory have been proposed and studied, such as invisibility cloaks^3^,^4^,^5^,^6^,^7^, electromagnetic concentrators^8^,^9^,^10^, carpet cloaks^11^,^12^,^13^, illusion devices^14^,^15^,^16^, flattened Luneberg lenses^17^ and high-resolution imaging lens^18^ and so on.

In this paper, we propose and experimentally demonstrate a novel illusion device that transforms a metallic cylinder into the Luneburg lens through the technique of transformation optics. Enclosing the metallic cylinder by the transformed lens, the light will be focused on one point after propagating around the metallic cylinder. To realize the required-anisotropic parameters of high permittivity and low permeability, we propose a kind of embedded split-ring resonators (SRR) to increase the permittivity of conventional SRR structures. In experiments, we fabricate and measure the transformed lens, which can guide the incident waves and focus on a spot after propagating around the center metallic cylinder, hence, the information of the object behind the obstacle can be guided around the obstacle and transmitted to infinity. The tested result is in consistence with both the theory and the simulations. We also observe that the fabricated transformed lens can work in about 200 Megahertz bandwidth.

## Results

### Theory and Analysis

**Figure 1:**
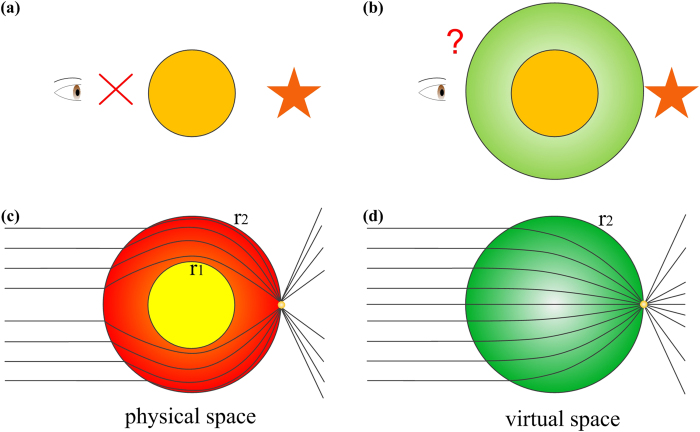
Illustration of transformed Luneburg lens. (**a**) When an obstacle is placed on the front of the eye, we cannot see anything behind the obstacle. (**b**) When the obstacle is enclosed by a dielectric lens, the things behind the obstacle can be observed. (**c**) Physical space. (**d**) Virtual space.

In real life, people may wonder whether we can see something behind an obstacle. Apparently, the answer is no, as shown in Fig. 1(a). However, if the obstacle is enclosed by a dielectric lens as illustrated in Fig. 1(b), things may happen in the other way. To make the object behind an obstacle visible, we take advantage of the super properties of inhomogeneous and anisotropic metamaterials to design a transformed lens, the principle of which can be found in Fig. 1(c,d). In the physical space, a metallic cylinder is enclosed by a transformed lens as shown in Fig. 1(c). Waves will be reflected once they access the surface of the metallic cylinder. However, when the metallic cylinder is enclosed by a circular-ring lens with inner radius *r*_*1*_ and outer radius *r*_*2*_, we expect that the waves will be guided and then propagate around the metallic cylinder. To design such a functional device by using the technique of transformation optics, an inhomogeneous cylindrical lens, Luneburg lens, exists in the virtual space, as shown in Fig. 1(d). Assume the distance between the center and focal point is *a*_*f*_ (namely the focal length). Then the material parameter of the lens with radius *r*_*2*_ is described as


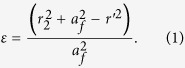


For example, if the focal length is chosen as *r*_*2*_, the focal point is located at the boundary of the cylindrical lens.

The transformation between the physical space and virtual space can be constructed as


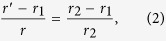


where *r’* and *r* denote the positions in virtual and physical space, respectively. Based on transformation optics theory, we can obtain the electromagnetic parameters for the transformed ring lens. Without loss of generality, here we consider *E*_*z*_-polarized plane waves since the TM mode is similar due to the dual principle^19^. For the case of TE-polarization, only *ε*_*z*_, *μ*_*r*_, *μ*_*φ*_ are interested. Hence, the electromagnetic parameters are given as follows:


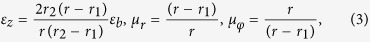


where 
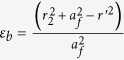
 and 
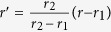
, and *a*_*f*_ represents focal length, which determines the position of focal point when the plane waves are incoming from the other side of circular ring lens. From equation (3), we can find that all three components of the transformed lens are gradient functions of the radius *r* making it difficult to control and manipulate the material property by metamaterials in reality. Here, we rescale the material parameters as follows,


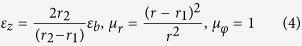


**Figure 2:**
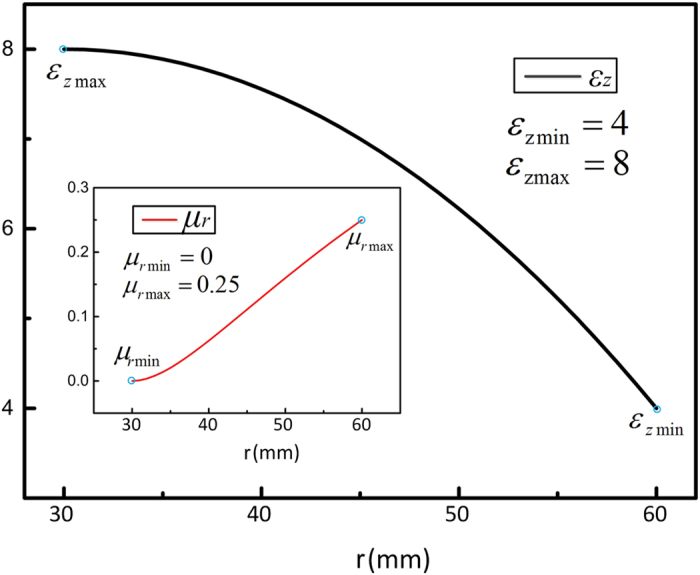
The parameter distribution of rescaled transformed lens when *a*_*f*_ =* r*_*2*_, *r*_*1*_ = 30 mm, *r*_*2*_ = 60 mm.

The medium parameters are illustrated in Fig. 2 when the geometrical parameters are chosen as *a*_*f*_ *=* *r*_*2*_, *r*_*1*_ *=* 30 mm, *r*_*2*_ = 60 mm, in which, *ε*_*z*_ ranges from 4 to 8 and *μ*_*r*_ ranges from 0 to 0.25.

**Figure 3:**
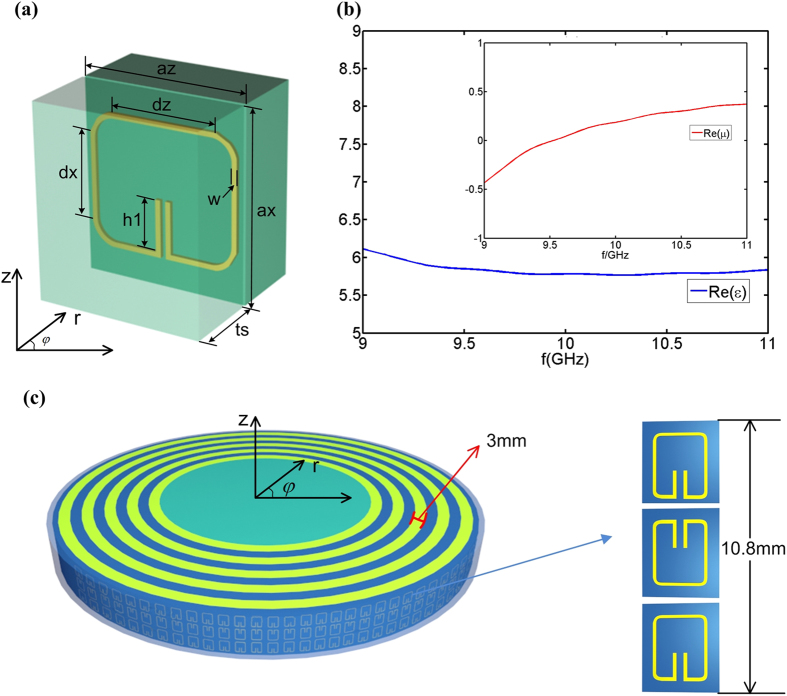
The details of embedded unit cells and effective material parameters. (**a**) The unit cell. (**b**) The effective material parameters of the eighth layer. (**c**) The illustration of transformed lens.

From the aforementioned discussion, the permeability of the transformed lens is below 1 and we know that SRR structure is such a magnetic resonator that can achieve low permeability. Nevertheless, the conventional SRR cannot satisfy the required high permittivity. Hence, we present a new embedded SRR to implement the above constitutive materials. Figure 3(a) gives the design in which the metallic SRR structure is embedded in the center of the dielectric cuboid with permittivity of 2.65. For the geometry, *t*_*s*_ is the thickness of one-layer medium and *a*_*x*_, *a*_*z*_ are the length and width of the unit cell respectively. *d*_*x*_, *d*_*z*_ are the length of metallic structure SRR with respect to *x* and *z* directions. *h*_*1*_ is the height of split gap of SRR and *w* is the line width of metallic structure. The thickness of metallic SRR structure is 0.035 mm, much less than that of the dielectric cuboid. The effective medium parameters of the embedded SRR structure in the eighth layer are shown in Fig. 3(b). We can find that the geometry parameters *d*_*x*_, *d*_*z*_ shift the frequency of electric resonance while *h*_1_ shifts the frequency of magnetic resonance. As a result, the electromagnetic parameters in other layers can be obtained by adjusting the geometry parameters *d*_*x*_, *d*_*z*_ and *h*_*1*_ at certain frequency.

**Figure 4:**
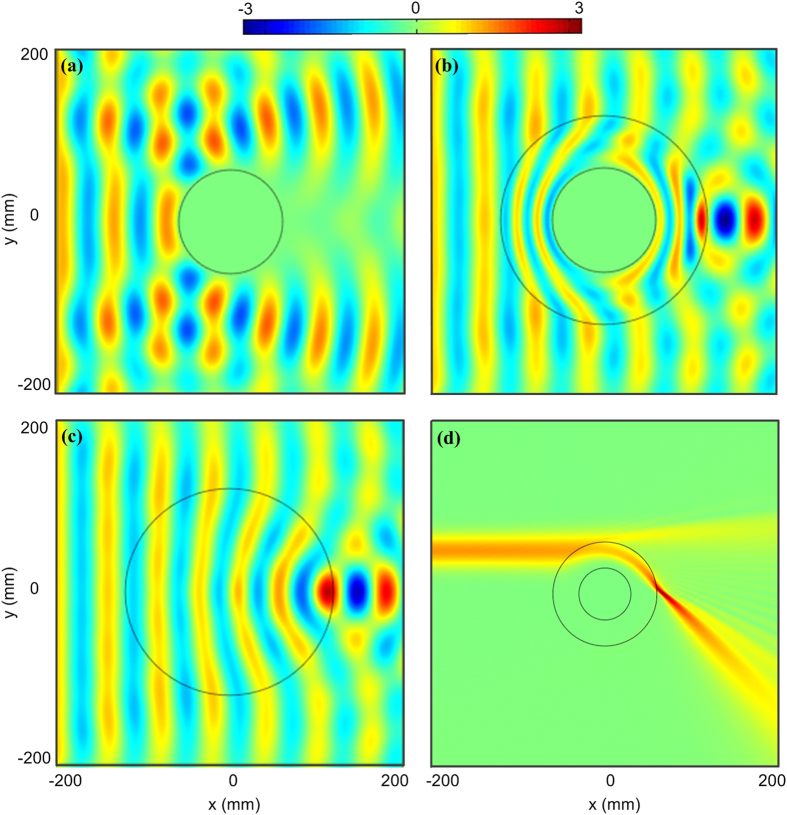
The simulated electric-field distributions. (**a**) The simulation results of a metallic cylinder. (**b**) The simulation results of a metallic cylinder enclosed by a transformed lens. (**c**) The simulation results of a Luneburg lens. (**d**) The simulated wave behaviors of a transformed lens at 100 GHz, which mimic some properties of the Einstein gravitational lens.

To verify the idea of this transformation lens, numerical simulations have been done by the aid of commercial software package COMSOL Multiphysics. The working frequency is chosen as *f*_*0*_ = 10 GHz. The radius of the center metallic cylinder is *r*_*1*_ *=* 30 mm, and the outer radius of the transformed lens *r*_*2*_ *=* 60 mm. The focal length is chosen as *a*_*f*_ *=* *r*_*2*_. Plane waves incident to a metallic cylinder without the transformed lens will be reflected and a shadow is observed in forward direction as shown in Fig. 4(a). After the metallic cylinder is enclosed by transformed lens, the waves will be redirected and then propagate around the metallic cylinder smoothly inside the lens, and finally focus on one spot when leaving the lens, as shown in Fig. 4(b). As a comparison, we plot the electric-field distributions of the original Luneburg lens in Fig. 4(c). It is easy to find that the field distributions outside the transformed lens and Luneburg lens are exactly the same (Fig. 4(b,c)), which verifies the theory of the transformed lens.

In the layout of the fabricated sample, the size of each unit cell along *r* direction is 3 mm (Fig. 3(c)). The geometrical parameters are *t*_*s*_ *=* 1.45 mm, *a*_*x*_ *=* 3.6 mm, *a*_*z*_ *=* 3.14 mm. Different layers share the same sizes of unit cells resulting in different numbers of the unit cells in different layers. The whole sample is divided into ten layers and the constitutive parameter in each layer is uniform since the material parameters change only in radial direction. Here, the size ranges of unit cell are given as follows. *d*_*x*_ is from 2.49 to 3.34, *d*_*z*_ is from 2.78 to 2.89, *h*_*1*_ is from 0.41 to 0.97, respectively. The whole fabricated sample is shown in Fig. 3(c), in which the height is 10.8 mm with three unit cells. Here we use yellow and blue to distinguish the number of layers. The metallic SRR structure is located in the middle of each layer.

**Figure 5:**
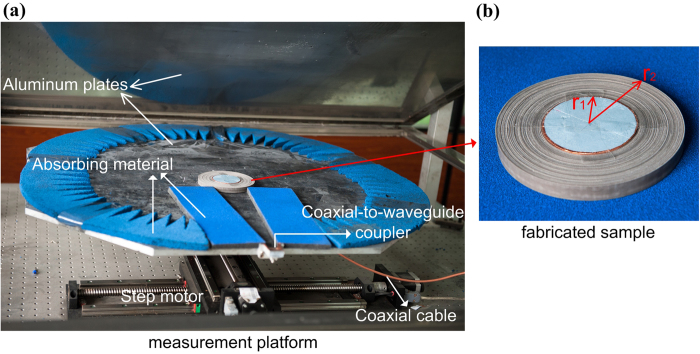
The test platform and fabricated sample.

As pointed out in above, the thickness of each layer is 3 mm. In real fabrication, it is very difficult to etch the metallic structures on the curving dielectric substrates. Hence, we employ 12 sub-layers to compose each layer. The thickness of each sub-layer is 0.25 mm. The metallic SRR structures are etched on the sixth sub-layer, which is in the center of each layer. The thickness of the metallic structures, 0.035 mm, is negligible, compared to the thickness of the dielectric layers. The fabricated lens is shown in Fig. 5(b). A copper cylinder is put in the center of the transformed lens serving as an obstacle thus the incident waves cannot penetrate into it. Here we use F4B as dielectric substrates with the relative permittivity of 2.65 and a loss tangent of 0.001.

Figure 5(a) gives the full view of our testing platform, a two-dimensional field-mapping setup (2D mapper), which is consist of two parallel aluminum plates, motors and vector network analyzer (Agilent N5230C). The distance between two parallel aluminum plates is set as 13.5 mm. The sample of the fabricated lens is placed on the center of the lower plate, which is mounted by a step motor to translate in two dimensions. A coax-to-waveguide coupler is employed as the feeding source to produce a narrow-width plane-wave beam. The total scanning region covers an area of 200*200 mm^2^. The feeding and detecting probes are connected to two ports of a phase-sensitive vector network analyzer via two coaxial cables.

### Experimental Results

**Figure 6:**
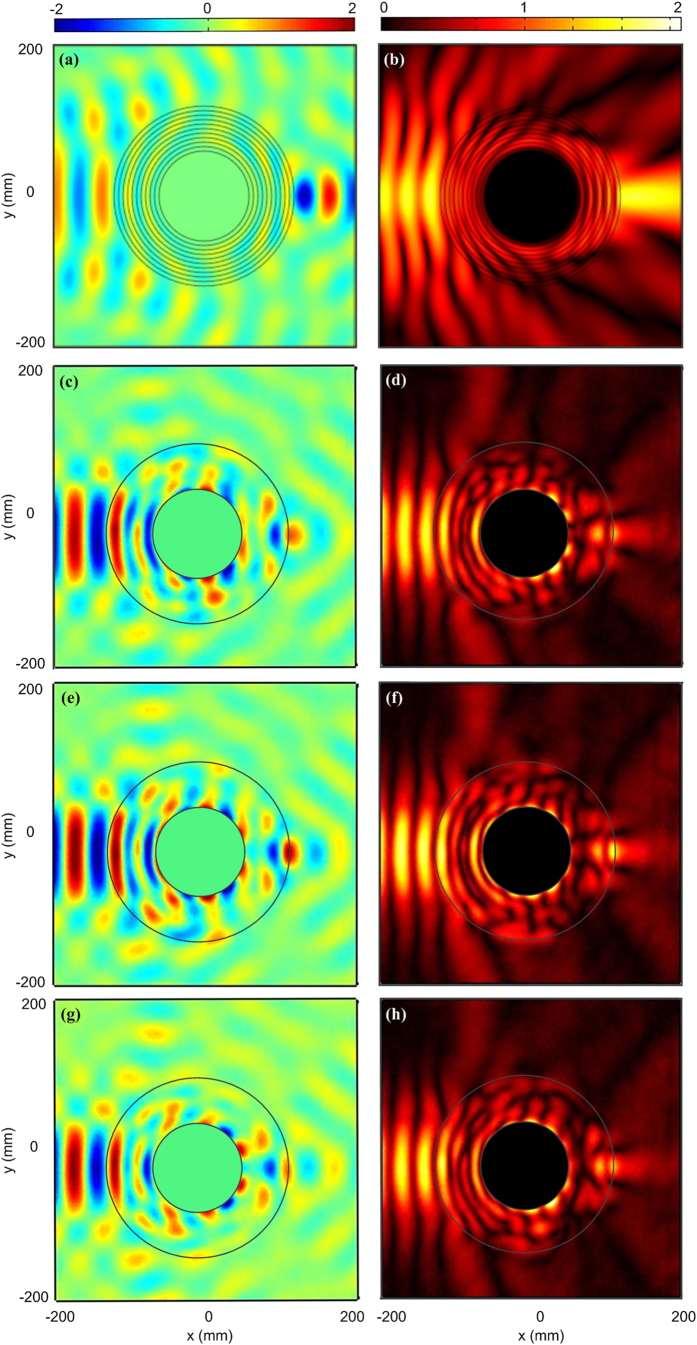
The simulated and measured real parts and amplitudes of electric fields. (**a**) The simulated real parts of electric fields with practical parameters. (**b**) The simulated amplitudes of electric fields with practical parameters. (**c**,**e**,**g**) The measured real parts of electric fields at 10 GHz, 9.9 GHz and 9.8 GHz, respectively. (**d**,**f**,**h**) The measured amplitudes of electric fields at 10 GHz, 9.9 GHz and 9.8 GHz, respectively.

Experiments are carried out to verify the implemented physical lens in our 2D mapper. Different from Fig. 4, another set of numerical simulations have been done with a narrow-width incident plane wave working at 10 GHz. The real part of electric field in the simulation is shown in Fig. 6(a), in which the practical retrieved parameters in each layer are considered. The outer and inner circles are the interfaces of the transformed lens. We can observe that the waves propagate around the center metallic cylinder and then focus on one spot. To observe the focusing effect more clearly, we also plot the amplitude distributions of electric field in Fig. 6(b). The field strength is obviously enhanced after propagating through the transformed lens and the focusing performance is validated. The measured real part of E-field of fabricated sample at 10 GHz is illustrated in Fig. 6(c), in which one focusing spot is observed near the transformed lens. The corresponding measured amplitude distributions of electric field are given in Fig. 6(d) and good focusing property is achieved. Figure 6(e,h) show the measurements of E-field distributions at 9.9 GHz and 9.8 GHz.

## Discussion

In above simulation and measurement results, the focus spots are located at the boundary of the transformed lens. Actually, we can choose the position of the focusing spot arbitrarily. If the focus length *a*_*f*_ < *r*_*2*_, the focal point will fall into the inside of transformed lens, while if *a*_*f*_ > *r*_*2*_, the focal point will fall outside of transformed lens. In the latter case, the waves will propagate around the center metallic obstacle and then focus on a spot outside the lens, similar to the light traveling in Einstein gravitational lens^20^. However, it is very difficult to investigate the light behavior of Einstein gravitational lens in experiment. Hence, the proposed transformed lens can be used to mimic some properties of the Einstein gravitational lens. Figure 4(d) shows the simulated light behavior around the transformed lens, which mimics some properties of the Einstein gravitational lens.

## Conclusion

In summary, we have proposed an illusion device that transforms a metallic cylinder into the Luneburg lens, in which the waves can be focused on one point after propagating around the metallic cylinder. In this manner, the information of the object behind an obstacle can be transmitted to the front obstacle, making the object visible. The required anisotropic parameters of high permittivity and low permeability are realized by a new embedded split-ring resonators (SRR) structure. Both the numerical simulations and experimental results of the fabricated transformed lens match the theoretical design very well working at about 200 MHz. The novel transformed lens can serve as an aid tool for the experimental study of Einstein gravitational lens with significant applications.

## Additional Information

**How to cite this article**: Xu, B. B. *et al.* Transmitting information of an object behind the obstacle to infinity. *Sci. Rep.* **5**, 13140; doi: 10.1038/srep13140 (2015).
